# More Questions Than Answers: A Narrative Review of the Epidemiology, Risk Factors, and Outcomes of Postoperative Atrial Fibrillation

**DOI:** 10.7759/cureus.103283

**Published:** 2026-02-09

**Authors:** Robert N Bilkovski, Jo Ann K LeQuang, Joseph Pergolizzi Jr

**Affiliations:** 1 Clinical Department, Native Cardio, Tampa, USA; 2 Scientific Communications Department, NEMA Research, Inc., Naples, USA; 3 Pain Medicine Department, NEMA Research, Inc, Naples, USA

**Keywords:** adult cardiac surgery, cardiac surgery, epidemiology of postoperative atrial fibrillation, noncardiac surgery, postoperative atrial fibrillation, racial disparities, sex-based disparities

## Abstract

Postoperative atrial fibrillation (POAF) lacks a consensus definition but is generally considered to be atrial fibrillation (AF) that develops following surgery. There is disagreement as to whether POAF must be de novo or whether a patient with a history of AF episodes can have true POAF. POAF has been more studied after cardiac versus noncardiac surgery, but this may be the result of less postoperative cardiac monitoring after noncardiac surgery. POAF can be clinically silent, paroxysmal, and self-terminating, or it may persist and have the potential to evolve into more chronic forms of AF. The pathogenesis of POAF has yet to be fully elucidated. A recent study indicates that postoperative atrial flutter may occur more often than previously thought and may be underestimated as a postoperative risk. Cancer surgeries have high rates of POAF, particularly following surgeries for lung, colorectal, gastrointestinal, and hematologic cancers. Among the best-known POAF risk factors are older age, male sex, White race, duration of surgery, comorbidities, and history of cardiac disease. The Black paradox of POAF finds that Black patients have more risk factors and more severe risk factors than White patients for POAF, yet Black patients are less likely to develop POAF than White patients. However, a Black patient with POAF is more likely to have a worse outcome than a White patient. A female paradox has also come to light, because women often have more risk factors for POAF than men but develop POAF less often; however, when POAF occurs in females, they can have severe outcomes. Urgently needed is a consensus definition of POAF to support epidemiologic and clinical work, and more widely used monitoring and surveillance tools to detect POAF when it does occur.

## Introduction and background

Postoperative atrial fibrillation (POAF) is a prevalent supraventricular tachyarrhythmia that emerges following surgery. Often defined as new-onset atrial fibrillation (AF) evident for the first time in the immediate postoperative setting, the lack of an expert consensus definition complicates efforts at epidemiologic understanding, quantification, and even diagnosis and treatment [1].

Some studies have defined POAF as any new-onset AF within 30 days following surgery; others narrow this postoperative window to a few days after surgery. There is disagreement as to whether a patient with pre-existing AF episodes can have true POAF after surgery [2, 3]; that is, AF episodes can occur after surgery in patients with a history of AF, but it is disputed whether this should be counted as true POAF. Asymptomatic AF, present in a subset of surgical patients, further renders this criterion of “new onset” imprecise. Some definitions apply the term POAF only to a supraventricular tachyarrhythmia in the postoperative period if that arrhythmia requires intervention [4], while others define POAF in part by a specific episode length of time, ranging from 30 seconds to 10 minutes [5, 6].

Mechanistic definitions and distinctions among POAF episodes are lacking because the pathogenesis of POAF is not completely understood. Genetics and an inflammatory response may play a more significant role in POAF than previously suspected [2]. Atrial flutter is emerging as an important postsurgical supraventricular tachyarrhythmia that may contribute to POAF; the incidence of postsurgical atrial flutter and its associated adverse effects may be underestimated [4, 7].

POAF has become an umbrella term for all types of perioperative AF, which has confounded efforts to better elucidate the mechanisms and risk factors for this condition. For example, there may be multiple types of POAF with different underlying mechanisms, risk factors, and probable outcomes. The current understanding of POAF is limited in part by the fact that most of the investigations into POAF have concentrated on cardiac surgery, although POAF may occur following other interventions. Continuous cardiac monitoring following cardiac surgery has helped better document POAF and postoperative atrial flutter after cardiac surgery, but continuous cardiac monitoring is less common after noncardiac surgery. For that reason, POAF following noncardiac surgery may be underestimated. This underscores the need for a consensus definition, as well as more routine postsurgical cardiac monitoring, even in patients not undergoing cardiac procedures.

Long thought to be a relatively benign, largely asymptomatic, and self-limiting condition, POAF today is understood to be associated with potentially serious long-term morbidity and mortality. Furthermore, POAF burdens the healthcare system in terms of costs and resource allocation [8]. Laudable efforts at risk stratification have not been entirely effective, although several risk assessment tools have been developed, and a risk-factor type assessment metric seems plausible [9].

POAF is associated with immediate as well as mid- and long-term consequences. POAF may be associated with longer-term risks for stroke, myocardial infarction, and the potential progression of AF, even to the development of chronic AF [2]. While the link between POAF and more persistent or permanent forms of AF remains speculative, there seems to be truth to the old saying that “AF begets AF,” since paroxysmal AF becomes permanent AF in about 25% of patients [10]. It is not known how frequently POAF becomes permanent AF. However, the emergence of POAF exposes underlying cardiac vulnerability to supraventricular tachyarrhythmias and cannot be dismissed as a temporary or self-resolving condition.

The aim of this narrative literature review is to explore what is currently known broadly about POAF epidemiology, its rate following cardiac and noncardiac surgery, risk factors, and observed racial, ethnic, and sex-based differences in POAF incidence and outcomes.

Methods

This is a narrative review of the peer-reviewed literature. Our inclusion criteria were articles that addressed the epidemiology of POAF, published in English, and published in the past 20 years. In some cases, we extracted other articles from the bibliographies of articles. Articles not relevant to our research goals, older than 20 years, or not in English were excluded.

We searched the PubMed database for “postoperative atrial fibrillation” (n=5,179) and “postoperative atrial fibrillation epidemiology” (n=1,904). Specific searches for “postoperative atrial fibrillation” combined with “race,” “sex,” “Black,” “Hispanic,” “White,” and “Asian” resulted in duplicate findings (no new results). Using the Cochrane database, a broad search was conducted for “postoperative atrial fibrillation,” yielding 10 results, but these articles addressed various treatment modalities rather than epidemiology and were excluded. We searched Google Scholar for “postoperative atrial fibrillation” and “postoperative atrial fibrillation epidemiology.” Bibliographies of relevant articles and guideline documents were also included. All searches were conducted in April and May of 2025. Due to the narrative review nature, formal quality assessment of clinical studies was not performed, since most of our search results were not clinical trials or randomized clinical trials. Because this is a narrative literature review rather than a systematic review with meta-analysis, we did not formulate a research question or restrict the search to only clinical trials or randomized clinical trials. Rather, we sought to provide a narrative overview of the broad subject of POAF epidemiology, focusing on what is known and what is not known to date.

## Review

The reported incidence of POAF varies widely, with the differences often attributed to any or several of the following: the nature of surgery (cardiac, thoracic but noncardiac, or non-thoracic and noncardiac), patient demographics (age, race, sex), how POAF is defined, and if and how the patient’s heart rhythm was monitored postoperatively. Monitoring techniques can play an under-appreciated role in POAF epidemiology, since POAF can be brief, clinically silent, and self-terminating without signs or symptoms. It is possible for a patient undergoing noncardiac surgery to experience POAF that goes completely undetected. Getting POAF episodes to “go on the record” had been very challenging in the era before continuous cardiac monitoring devices and may still explain why the POAF rates following noncardiac surgery are remarkably low. The plausibly multimechanistic nature of POAF may also contribute to variations in POAF rates and outcomes. The studies of POAF are heterogeneous, which can further complicate interpretation.

Clinical presentation 

POAF is paroxysmal, often asymptomatic, occurs two to four days post surgery, is intermittent, and occurs in brief episodes that are difficult to detect clinically, but appear on monitors [3, 8]. The advent and practicality of implantable loop recorders and other monitoring systems have helped better quantify POAF and subsequent AF that may have otherwise eluded detection.

By many definitions, POAF must be a new-onset AF in the perioperative setting; however, some consider the exacerbation of a preexisting arrhythmia to also be POAF [11]. It may be difficult to ascertain if POAF truly occurs de novo, since paroxysmal AF apart from the perioperative setting can be asymptomatic and resolve on its own. In all cases, POAF occurs because there is an underlying substrate to support it, thus revealing cardiac vulnerability, which may subsequently allow other forms of AF.

The stress of cardiac or even noncardiac surgery on a patient who has experienced prior episodes of AF may set the stage for POAF. It is estimated that about 2.5% of the general population has paroxysmal AF that may resolve spontaneously [12]. Atrial flutter can occur concomitantly with AF or may precede the development of AF; the interrelationship between atrial flutter and AF is not well studied [13]. Atrial flutter appears to be much less prevalent, but it can be associated with morbidity and increased healthcare costs [14]. The prevalence of atrial flutter in the United States in all settings has been stated as 88 per 100,000 patients [15]. Atrial flutter and AF can present as comorbid conditions; it has been estimated that 80% of people with atrial flutter have episodes of AF, and approximately 20% of people with AF have atrial flutter as documented on a 12-lead electrocardiogram (ECG) [16]. The interplay between atrial flutter and AF, particularly in the context of POAF, warrants further study.

In many instances, POAF will spontaneously convert to sinus rhythm, which is likely what has contributed to the mistaken notion that POAF is benign [17]. The long-term consequences of postoperative supraventricular tachyarrhythmias warrant further investigation.

Pathogenesis of POAF

Postoperative inflammatory response, sympathetic activation, and cardiac ischemia have been implicated in POAF [3], which must be considered a unique rhythm disorder, because it is a specific arrhythmia defined by the postoperative setting, where postoperative stress may stimulate the adrenergic system, resulting in higher-than-normal levels of catecholamines circulated in response to pain and/or inflammation [18]. The differences in pathogenesis between POAF and other forms of AF remain to be more fully elucidated.

The pathogenesis of POAF exceeds the scope of this review, but it is clear that POAF and other forms of AF involve an atrial substrate vulnerable to arrhythmic activity [3]. Surgery may expose this vulnerability because it can lead to atrial stretch or other deformations, which might increase intravascular overload and contribute to POAF [18].

Treatment of POAF

At present, the optimal preventive therapy for POAF is beta-blockade, ideally commenced before surgery [3]. Amiodarone is sometimes used in combination with beta-blockers. In an observational study of 454 coronary artery bypass surgery patients, amiodarone was shown to lower the incidence of POAF or delay its onset [19]. Anti-inflammatory therapeutic agents may also be helpful [3].

There are two main approaches to treating all forms of AF, including POAF. Rate control attempts to slow the ventricular response to a rapid atrial rhythm, while rhythm control attempts to convert the AF back to a normal sinus rhythm. In most cases, POAF is treated with rate control, but conversion may be used if rate control is inadequate or there are signs of hemodynamic instability [3]. A retrospective observational study of 6,435 patients undergoing coronary artery bypass and/or valve procedures reported that 33.8% experienced POAF, of whom 26.4% required electrical cardioversion to restore sinus rhythm [20]. Patients with heart failure are at elevated risk for POAF following cardiac surgery, and in this population, pharmacologic rhythm control or electrical cardioversion were associated with a lower AF burden in the first seven days and a more rapid restoration of sinus rhythm [21].

POAF after cardiac surgery

POAF is the most common complication following cardiac surgery and can have long-term adverse consequences [22]. POAF occurs in approximately 35% of all cardiac surgery cases [23], with even greater incidences up to 47.3% when coronary artery bypass grafting (CABG) is combined with valve repair or replacement [24]. The highest rates of POAF occur in conjunction with valve repair/replacement procedures; isolated CABG procedures have a lower incidence of POAF [24]. A study of 11,239 consecutive CABG patients reported an unadjusted incidence of new-onset POAF at 29.5%, with most arrhythmic episodes of several hours’ duration [1].

During cardiac surgery, the heart may be manually manipulated and instrumented, cardiac pressures can become elevated, and a localized inflammatory response can occur. Any or all of these conditions might trigger an arrhythmic response that may manifest in POAF hours to days after surgery. The frequently observed self-termination of POAF may be attributable to the resolution of the local injury and inflammation [25].

The incidence of POAF following cardiac surgery varies by the type of surgery. In a study of 153 patients who underwent CABG with cardiopulmonary bypass compared to 76 without bypass, those on cardiopulmonary bypass had higher rates of POAF (37.6%). In particular, new cases of POAF were greater among those with bypass than those not on bypass (21.8% vs. 15.7%, p=0.031) [26]. Myocardial revascularization surgery combined with valve surgery has a reported incidence of POAF as high as 80% [27]. 

POAF after noncardiac surgery

Approximately 7% of patients having major noncardiac surgery develop POAF [28]. POAF following noncardiac surgery is not rare, and the limited data show a wide range of incidences, likely due to the heterogeneous surgeries, diverse patient populations, and lack of standardized electrophysiology protocols to monitor patients after surgery [29, 30]. POAF rates range from <1% to 15% for all types of noncardiac surgery, but certain noncardiac surgeries, such as colorectal procedures and liver transplant, are associated with higher rates of POAF than other noncardiac operations for unknown reasons [31]. In a single-center study of 571 consecutive colorectal surgery patients with no history of AF, 6.6% developed POAF; however, in this study, POAF was defined as AF within 30 days of surgery [32]. A systematic review of POAF incidence after general surgery (13 studies, n=52,959) found 10.9% developed POAF after general surgery, with the highest rate of POAF in esophagectomy (n=376), which had a weighted average of 17.7% cases of POAF [33].

In a study of 857 adult patients with no history of AF who underwent orthotopic liver transplantation, 10.4% developed POAF following surgery [34]. In this study, POAF was limited to only new-onset arrhythmia and was associated with in-hospital mortality (7.2% vs. 2.8%). One-year mortality in this study was 22.4% for POAF patients compared to 8.3% for those without POAF. Another study of liver transplant patients found the duration of POAF was not associated with long-term survival [34]. A study of 461 liver transplant patients reported that POAF occurred in 10.2% of patients in the first three postoperative days and was associated with an eight-fold elevated risk of thromboembolic events [35]. A retrospective database study of 1,011 liver transplant patients found the incidence of POAF to be approximately 10%, but some of the included patients had a history of AF; in fact, a preoperative history of AF was determined to be the strongest predictor of POAF in this study [36].

In contrast to cardiac surgery patients, noncardiac surgery patients who develop POAF have a higher risk for complications, such as bacterial pneumonia and congestive heart failure. It is speculated that following noncardiac surgery, POAF tends to occur in the sickest patients, who are more susceptible to these complications [25]. As the surgical population expands to include a greater proportion of older and frail patients, the incidence of POAF after noncardiac surgery could reasonably be expected to increase [25].

POAF after noncardiac surgery is not necessarily symptomatic. In an observational study of 99 non-cardiothoracic surgery patients who developed POAF, only 30% reported symptoms. However, patients with asymptomatic POAF had statistically similar outcomes as those patients who had symptomatic POAF [37]. Considering the number of noncardiac surgeries compared to cardiac procedures, the incidence of POAF after noncardiac surgery is likely to be of considerable and underappreciated significance.

POAF and cancer surgery

Oncologic surgery patients may be at elevated risk for POAF, and the bidirectional association between AF and cancer is currently a subject of active research interest [38]. Cancers most closely associated with AF are lung, colorectal, gastrointestinal, and hematologic cancers [38].

In a prospective study of 400 consecutive cardiac surgery patients at a single center in Cyprus, 16.5% developed POAF, with cancer emerging as a significant predictor of POAF (odds ratio 3.852, p=0.004) [39]. Following cancer ablation procedures, POAF could be associated with higher rates of surgical morbidity and postsurgical mortality compared to patients without POAF. In a study of 26,817 head and neck cancer patients who underwent tumor ablation reconstructed with microvascular free tissue transfer, those with POAF were at higher risk for flap failure. In POAF patients with pre-existing AF, the all-cause mortality rate was significantly greater (p<0.001) than for those POAF patients with no history of AF [40]. Further study is warranted into the association between cancer surgery and POAF.

Early versus late POAF

In studies of POAF, the terms “early” versus “late” POAF are sometimes used, but without a universally accepted and consistent definition. Both "early" and "late" apply to the temporal relationship between surgery and the onset of POAF. The timing of POAF onset may play a role in outcomes.

In the RACE-V study, 98 consecutive cardiac surgery patients were monitored continuously by an implanted loop recorder for 2.5 years following surgery to differentiate between the burdens of early versus late POAF [41]. In this study, “early POAF” was defined as any AF episode(s) that occurred in the first 90 days after surgery, while “late POAF” was defined as episodes that occurred after that 90-day window. Early and late POAF had some association, because late POAF occurred mainly in those patients who had previously had early POAF. The study evaluated POAF burden, defined as the number of episodes of POAF, their duration, and the proportion of time spent in POAF over a given time period. The RACE-V prospective cohort study found a significant association between POAF burden overall and the duration of the longest individual POAF episode; that is, longer episodes increased POAF burden. A higher early POAF burden was associated with a greater likelihood of late POAF episodes. An important finding of the RACE-V study was that early POAF burden rather than POAF incidence may be a more accurate predictor of late POAF. Moreover, late POAF burden is suggestive of atrial remodeling and the concomitant elevated risk of adverse effects [41]. Subgroups of patients from this study were monitored for 2.5 years and then analyzed using electrocardiography (ECG) and laboratory biomarkers for the occurrence of POAF. Among 87 patients with no preoperative history of AF, 47% had early POAF and 25% had late POAF. Early POAF was so closely associated with late POAF that 90% of those with late POAF had reported early POAF [42]. In patients with a preoperative history of AF, 67% developed late POAF. Thus, in cardiac surgery patients with or without a history of AF, the incidence of late POAF was high [42]. In this context, it should be noted that in real-world clinical practice, late POAF may not always be linked to an earlier surgery, such that late POAF may be under-estimated and considered to be de novoAF.

In a study of 79 cardiac surgery patients with no history of AF, late POAF occurred in 67% of those who had early POAF and 37% of those who did not have early POAF. This study reported early POAF as a predictor of late POAF, even among patients with no preoperative history of AF. In this connection, it is important to note that early POAF was defined as occurring in the first five preoperative days [43]. Consensus definitions of POAF, as well as early and late forms of POAF, will greatly aid quantitative assessments.

Time of day may play a role in early versus late POAF. Early POAF, particularly episodes that occur in the first few days following surgery, show a more uniform distribution over time of day, while late POAF tends to peak during daytime hours, mostly between 6 a.m. and 6 p.m., with most occurrences between 8 and 11 o’clock in the morning [43]. This pattern implicates adrenergic triggers for late POAF, making this supraventricular tachyarrhythmia mechanistically distinct, yet associated with early POAF [43].

The difference between early and late POAF might also be framed as POAF versus AF recurrence. AF recurrence was studied in a systematic literature review and meta-analysis, reporting that 28.3% of those who experienced POAF in the immediate postoperative period had AF recurrence in the first four weeks after discharge, as detected on noninvasive monitoring [44]. Among those surgical patients monitored continuously for over the next two years with implanted loop recorders, AF was detected in 61% to 100% of patients, with 40% to 93% of all episodes asymptomatic and thus likely to be clinically silent and under-reported [44]. In a study of cardiac surgery patients at risk of stroke but with no history of AF, continuous monitoring by an implanted loop recorder found that asymptomatic episodes of AF increased significantly after discharge from the hospital and were only detected due to continuous monitoring [30].

Morbidity and mortality following POAF

Long-term consequences of POAF are not well studied. In a study of 2,985 CABG patients, 18.5% developed POAF, which was a predictor of long-term mortality (hazard ratio 1.21. 95% confidence interval, 1.12 to 1.32) over a follow-up of an average of six years [45]. Following cardiac surgery, POAF is independently associated with in-hospital mortality, recurrence of AF in the future, and long-term mortality [46-49]. Among CABG patients, POAF emerged as a significant independent predictor of long-term newly developed AF (hazard ratio 4.99, p=0.004) [46]. A retrospective database study of 994 CABG patients stated that POAF occurred in 16% of the cohort and was associated with long-term stroke, in-hospital mortality, and longer hospital stays; four- and five-year survival was significantly decreased in POAF patients [47]. On the other hand, POAF was negatively associated with myocardial infarction in this study [47].

The widespread use of implantable loop recorders has greatly aided research into recurrent AF following POAF. In a study of 42 cardiac surgery patients, 30 of whom developed POAF, continuous monitoring using an implantable loop recorder found 76% of those with POAF had recurrent AF episodes in the first year following surgery, and 30% had recurrences beyond one year [50]. In a community-based study of 603 cardiac surgery patients, POAF was a predictor of recurrent AF, which, in turn, was associated with higher mortality [49].

A Swedish nationwide cohort study examined data from 35,329 CABG patients, of whom 30% developed POAF. With a median follow-up of seven years, it was found that 6.7% of those with POAF had an early recurrence of AF. Early recurrence of AF was associated with increased hospitalization for heart failure and major bleeding events, but not with all-cause mortality [51].

Following cystectomy, POAF occurs at a rate of 2% to 8%; a retrospective analysis of 4,345 cystectomy patients found that those with POAF had a higher cumulative incidence of cardiovascular adverse events in the first year after surgery than those who did not have POAF (24.8% vs. 10.9%, p=0.007) [52]. In a study of gastrectomy for malignancy patients, those who developed POAF had a higher rate of cardiovascular events in the first year after surgery [53]. In a qualitative systematic literature review of POAF in noncardiac surgery patients, POAF increased the long-term risk of mortality and increased the risk of stroke four-fold (odds ratio 4.05) [54]. A systematic review and meta-analysis involving 14 studies, including >3.5 million surgical patients, reported that POAF occurred in less than 1% of cases, but of the patients who developed POAF, 1.5% had a stroke compared to 0.4% who did not develop POAF. Pooled analysis showed POAF in this population increased the relative risk (RR) of stroke by a factor of 2.5. Stroke risk was significantly greater among POAF patients who had nonthoracic noncardiac surgery (RR 3.09) compared to those who underwent thoracic surgery (RR 1.95), p=0.01 [55].

A population-based study of 32,160 noncardiac surgery patients with a history of nonvalvular AF prior to surgery evaluated patients based on a composite endpoint of 30-day mortality, stroke, transient ischemic attack, or systemic embolism. The 30-day risk for this composite endpoint was 4.2% [56].

In a study of 752 consecutive patients undergoing cancer surgery at a single center, 10.2% developed POAF and, of this subset (n=77), 31% experienced recurrent AF compared to <1% of the patients who did not develop POAF [57]. Although often asymptomatic, recurrent AF is associated with elevated risk of stroke and all-cause mortality in these cancer patients [57, 58]. At three years, it was found that POAF could be correlated with the incidence of cancer death; in other words, POAF could be associated in this population with the recurrence of malignancy [59].

A retrospective review of 1,031 patients with no history of supraventricular tachycardia (AF or atrial flutter) and who underwent cardiac surgery from 2010 to 2018 found that 43% had early POAF, which was described as "typically transient," lasting less than 48 hours in 72% of patients, and spontaneously converted back to sinus rhythm at hospital discharge in 91% of patients. After 4.7 ± 2.4 years of follow-up, late or recurrent AF was observed in 14% of patients, with a median time of 4.4 years after surgery to AF recurrence. Patients with early POAF were significantly more likely to develop late POAF (23% vs. 6%, p<0.001) [60]. In patients with early POAF lasting >48 hours, the hazard ratio for late POAF was 5.9 [60].

Risk factors

The currently known risk factors for POAF include older age, male sex, White race, cardiac as opposed to noncardiac surgery, duration of surgery, comorbidities, history of cardiac disease, and a range of postoperative complications, such as sepsis [9, 33]. Age is the most robust and reliable risk factor for POAF and applies to all races [61]. Patients ≥ 72 years of age have a five-fold increased risk of POAF after surgery compared to patients ≤ 55 years of age [62].

Many common comorbidities are thought to elevate a surgical patient's risk for POAF, such as hypertension, history of myocardial infarction, congestive heart failure, and renal failure [4, 18]. Inflammation may also be a risk factor for POAF, which has been associated with the biomarker of higher levels of C-reactive protein [63].

In cardiac surgery patients, serum bone morphogenetic protein 10 (BMP10) serves as an AF marker and suggests the risk for major adverse cardiovascular events (MACE), including stroke and mortality. In a substudy of 147 cardiac surgery patients taken from the RACE-V study, BMP10 was shown to accurately represent the patients’ prior history of persistent AF and was associated with an increased risk for late POAF, that is, AF occurring 90 or more days after surgery [64]. In a study of 2,104 cardiac surgery patients, risk factors for POAF found on multivariable analysis included older age, White race or non-Hispanic ethnicity, history of heart failure, and history of hypothyroidism [24]. In this study, diabetes mellitus type 2, history of myocardial infarction, and rheumatic heart disease did not emerge as risk factors for POAF [24].

Obesity, obstructive sleep apnea, and sleep-disordered breathing may also elevate a surgical patient's risk of POAF [65]. Obesity, whether measured in body mass index (BMI) or body surface area (BSA), is an established risk factor for POAF following CABG procedures [66]. BSA may be a more accurate metric than BMI in this connection, although further study is needed. People with a BSA around 2.0 m^2^ typically have a large left atrium and abnormal intrathoracic pressure, which can further disrupt atrial electrophysiology [67]. Obese patients face a significantly higher risk for POAF compared to non-obese patients following cardiac procedures (p=0.005) and a concomitantly and significantly greater risk of stroke (p<0.0001), respiratory complications (p<0.00001), and 30-day mortality (p=0.05) [68]. In patients >50 years of age, obesity is an independent risk factor for POAF after CABG procedures [69]. Obesity is also a recognized risk factor for POAF after noncardiac surgery, but has not been thoroughly studied [70]. Increased rates of obesity in the United States and other parts of the world are likely to contribute to an increase in POAF [71].

A newly recognized risk factor for POAF is sleep-disordered breathing, which, in an unadjusted analysis, was associated with perioperative AF following cardiac surgery. In a study of 190 cardiac surgery patients, those with a BMI >32 kg/m^2^ and sleep-disordered breathing increased their chances of POAF by 15% [72]. Sleep-disordered breathing is prevalent among cardiac surgery patients and has been associated in the NU-SLEEP trial with POAF [73].

While some risk factors for POAF apply to all types of surgeries, risk factors for POAF following noncardiac procedures include mitral regurgitation in the population of critically ill patients [74]. In a study of 2,588 patients undergoing noncardiac thoracic surgery, POAF developed in 12.3%, and independent risk factors included male sex, older age, history of congestive heart failure, history of arrhythmia, history of peripheral vascular disease, resection of mediastinal tumor or thymectomy, lobectomy, bulbectomy, pneumonectomy, esophagectomy, and intraoperative transfusions [75].

A study evaluated 2,104 patients before and after scheduled cardiac surgery to assess for POAF, which occurred in 28.1% of patients who had an isolated CABG procedure, in 33.7% who underwent an isolated valve repair or replacement, and in 47.3% of those who had a CABG procedure plus valve repair or replacement [24]. Risk factors for POAF in this population were identified as older age, White race, history of heart failure, and a history of hypothyroidism. No medications were associated with an elevated risk of POAF [24].

Genetics is being explored with respect to POAF risk. Seventeen independent signals from 14 genomic sites have been identified as risk factors for AF, and it is suspected that they play a role in POAF [61]. Factors capable of causing structural damage to the heart or electrical “rewiring” of the atria could also be considered POAF risk factors; such factors could encompass certain modifiable factors, such as sedentary lifestyle, smoking, and unhealthy diet [61].

Race and ethnicity

White patients are at elevated risk for POAF following cardiac procedures; a study of 5,823 patients undergoing isolated CABG procedures reported POAF in 32.4% of White patients compared to 21.3% of non-White patients [76]. The Epidemiology, Practice, Outcomes, and Costs of Heart Failure (EPOCH) study reported that among heart failure patients, Black patients had a significantly lower incidence of AF (as distinct from POAF) than White patients (19.7% vs. 38.3%, p<0.001) [77] and the Atherosclerosis Risk in Communities (ARIC) study found the same thing with Black patients having markedly lower rates of AF than non-Black patients (2.7% vs. 5.0%) [78]. It is paradoxical that Black patients enter surgery with more and more severe risk factors for POAF (such as hypertension, obesity, diabetes, and congestive heart failure) than White patients, but have lower rates of POAF [7].

Initially, the POAF racial paradox was explored through the framework of healthcare disparities, such as income, access to healthcare, health insurance, and bias. The Multi-Ethnic Study of Atherosclerosis (MESA) study monitored 1,556 people (41% White, 25% Black, 21% Hispanic, 14% Chinese; 51% female) both clinically and by ambulatory ECG monitoring device. When the study participants were followed clinically, the rate of AF was lower in Black patients than in White patients, regardless of comorbidities or risk factors. However, when AF was detected solely by the monitor, the rates of AF were similar among racial groups. This suggests that some element of clinical bias may intrude on racial data related to AF [79].

While research in the field of racial differences in POAF tends to focus more on Black patients than other minorities, minority patients taken collectively tend to have more comorbid conditions than White patients. As a group, minority patients are more likely to be on dialysis or have a history of diabetes or stroke. Furthermore, minority patients are often of lower socioeconomic status and may have limited or no health insurance and less access to healthcare [80]. Yet minority patients have a significantly lower rate of new POAF than White patients (19.5% vs. 29.5%, p=0.02) as reported in a study of 1,218 patients undergoing isolated CABG. In this study, 215 of the total 1,218 patients (17.7%) represented a minority [80].

The left atrial volume index emerges as an interesting variable in this context. Among White patients only, a higher left atrial volume index has been associated with POAF (31.3% vs 29.3%), but left atrial volume has no such association in non-White patients [80]. During cardiac surgery, non-White patients used greater amounts of intraoperative blood products than White patients and trended toward greater use of postoperative blood products [80]. Although minority patients had lower rates of POAF following CABG surgery than White patients, they required a longer median stay in intensive care and longer hospital lengths of stay [80]. When minority patients were taken to include Black, Hispanic, Asian, and Native American patients, they had a significantly lower rate of POAF after isolated CABG surgery than White patients (19.5% vs. 29.5%, p=0.02) [80]. This study of 1,218 patients suggests that the left atrial volume index could be a predictor of POAF in White patients, but not all groups [80]. An explanation for this has been offered, which speculates that the geometry of the left atrium is more influential than left atrial volume alone [81]. For example, nonoperative AF can enlarge the left atrium, so left atrial geometry (volume plus diameter) may indicate an underlying supraventricular tachyarrhythmia, which may not always manifest as left atrial volume alone.

While Black patients undergoing CABG procedures have lower rates of POAF than similar White patients, when POAF does occur, they have worse outcomes to the point that POAF is a significant predictor of mortality in Black CABG patients [82]. In other words, POAF among CABG patients is less frequent among Black patients than White patients, but more deadly. In contrast, White people have fewer risk factors for AF, but higher rates of AF and POAF than minorities [76, 83].

In a propensity-matched study of Black and White patients undergoing CABG procedures, Black patients had a significantly higher rate of major adverse cardiovascular and cerebrovascular events (MACCE) than White patients (7.6% vs. 3.7%, p=0.013). Among the MACCE predictors for Black patients were POAF as well as creatinine level, chronic obstructive pulmonary disease, and a left-ventricular ejection fraction <50% [84]. Risk predictors for MACCE among White patients did not include POAF [84]. In a retrospective analysis of the Society for Thoracic Surgeons database from 2011 to 2018, the records of over 1 million CABG surgery patients were analyzed, with the result that Black patients were determined to have the highest rates of preoperative comorbidities compared to other races and Black patients had significantly higher overall mortality compared to White patients (2.76% vs. 2.19%, p<0.001) [85]. Yet Black patients are not as likely as White patients to develop POAF following CABG surgery [86, 87]. For both Black and White patients, common risk factors for POAF from a cohort study (n=1,215) were listed as follows: older age and history of hypertension, heart failure, and stroke. However, the other risk factors of obesity, smoking, diabetes, dialysis, history of myocardial infarction, and prior stroke were stronger risk factors for Black patients versus White patients [87].

Genetics may provide more and better answers to this racial paradox. The ARIC evaluated the risk of AF and other conditions in 14,419 participants from several races [78]. The ARIC found that while Black patients had a lower risk of AF than White patients, another study determined that those Black patients with a higher proportion of European genetic ancestry had a greater risk of AF compared to Black patients with less genetic European ancestry. In fact, for each 10% increase in European ancestry of a Black participant, the hazard ratio for AF increased 1.17 [88]. Evidence that a genetic explanation may help explain the racial differences was found in a geographical study of POAF incidences among 4,657 patients following CABG surgery. The rate of POAF following CABG surgery was 33.7% for the United States, 36.6% for Canada, 34.0% for Europe, 31.6% for the United Kingdom, 41.6% for the Middle East, 17.4% in South America, and 15.7% in Asia [89].

There is a paucity of POAF studies among Asian patients. A study of 711 cardiac surgery patients in Thailand found 31% of these patients developed POAF over a median 10-day hospitalization [90]. Most of these patients underwent isolated valve surgery (69%), but others had isolated CABG procedures (25%) or concomitant CABG plus valve surgery (6%) [90]. In a prospective single-center study from China intended to help better quantify the rate of POAF in Chinese cardiac surgery patients, 266 consecutive patients with coronary artery disease who had isolated CABG surgery were enrolled, excluding those with a history of AF. In this study, the incidence of POAF was 47%. When cohorts with and without POAF were compared, those with POAF were older, more likely to have renal dysfunction, and were more likely to be smokers [91]. South Asian people, including those from India, Pakistan, Sri Lanka, Bangladesh, and Nepal, compose approximately 20% of the world’s population and have a relatively high prevalence of hypertension, diabetes, metabolic syndrome, and obesity. Despite these comorbidities and high rates of ischemic stroke, they have low rates of AF for reasons that remain unclear [92]. In a study from Singapore of 2,168 Chinese, Malay, or Indian patients undergoing CABG or valve surgery with cardiopulmonary bypass, POAF occurred in 17.3%, with the peak occurrence at 72 hours post surgery. In addition to risk factors identified in other studies, Chinese ethnicity was a more significant predictor of POAF than Indian ethnicity (odds ratio 2.09, p=0.003), and Malay ethnicity was a more significant predictor of POAF than Indian ethnicity (odds ratio 2.43, p=0.002). On the other hand, the risk of POAF was statistically similar for Chinese and Malay patients [93].

Among Black and White pacemaker patients, AF detection over 3.7 years found that Black patients had significantly fewer occurrences of AF than White patients (21.4% vs. 25.5%) but higher rates of atrial flutter [94]. 

Atrial flutter 

Atrial flutter is a supraventricular tachyarrhythmia distinct from AF, characterized on ECG by a regular sawtooth pattern indicative of high-rate, albeit organized, atrial activity. The regularity of the rhythm indicates a re-entry circuit. The rapid atrial beats do not all conduct down to the ventricles, so the ventricular rate is rapid but slower than the atrial rate. Like AF, atrial flutter may be asymptomatic, paroxysmal, resolve on its own, or it may persist and even transition into AF. The incidence of postoperative atrial flutter is not well known and, in many cases, is not even studied as its own specific arrhythmia. In some clinical studies, atrial flutter is grouped with other forms of POAF or AF [7].

The mechanism of postoperative atrial flutter is known; it is a macro-reentrant right-atrial tachycardia that can occur following surgical incision of the right atrium [95]. In a study of 67 consecutive patients who had undergone mitral valve surgery and presented with some form of supraventricular tachyarrhythmia following surgery, the right atrium was the point of origin for postoperative atrial flutter in 56% of cases, presumably because reentry circuits are found more frequently in the right atrium [96].

Atrial flutter has not been associated with the known AF risk factors of hypertension, obesity, diabetes, or left atrial volume and/or geometry. Like AF and POAF, it can be a challenging arrhythmia to study since episodes are rare, brief, and can be asymptomatic. Some early studies have found that while Black patients have a lower incidence of AF than White patients, they have a higher rate of atrial flutter [94, 97]. In a database study of 13,967,949 patients of multiple ethnicities followed over the course of 3.2 years, Black patients were shown to have a lower risk of AF than White patients (hazard ratio 0.84) but a higher risk for atrial flutter (hazard ratio 1.09) [97]. A study of 101,773 Black and White pacemaker patients confirmed that Black patients were at a lower risk for AF (hazard ratio 0.91) but at a higher risk for atrial flutter (hazard ratio 1.29) compared to White patients, based on the continuous monitoring data from the device [94].

Preoperative atrial fibrillation

Despite the prevalence of POAF and its potential consequences, there is limited research on the important topic of the relationship between POAF and pre-existing AF [98]. POAF demonstrates the presence of a viable and conducting atrial substrate, even if the patient has never yet experienced AF. 

A retrospective database study compared noncardiac surgery patients with and without a prior history of AF and concluded that preoperative AF is independently associated with postoperative adverse outcomes: mortality, heart failure, stroke, but with a reduced risk for myocardial infarction. These results were consistent across sex, race, and type of surgery [99].

Timing

In a retrospective single-center study of 53,387 surgery patients, POAF occurred in 1.1% of the cohort and, on average, 3.4 ± 2.6 days after surgery [63]. In another study, POAF was found to occur most frequently on the second postoperative day [89]. The timing of POAF has led to studies that differentiate POAF in the first 18 hours after surgery compared to POAF that occurs 2 or more days after surgery, naming these Phase 1 and Phase 2, respectively [100]. It has been reported that in cardiac surgery patients, the risk factors for POAF vary by phase, with obesity and White race risk factors only in Phase 2. In this study, Phase 2 commenced at 48 hours after surgery and extended for seven days. Other risk factors, such as older age and mitral valve procedures, were similar in both phases [100].

Sex

AF, distinct from POAF, has a higher incidence in men than women in most geographical regions, across all income brackets, and in all races and ethnicities [61]. In North America and Europe, men are more likely to develop AF, although women and men share a similar lifetime risk for AF, a fact that can be explained by the shorter life expectancy of men [101]. There may also be a “female paradox” with POAF, because a large database study of 21,568 cardiac surgery patients at two hospitals reported, after controlling for other factors, that women were less likely than men to develop POAF but, if they did, women had a higher risk of long-term mortality than men [102]. This has also been termed the “sex disparity” [103].

At baseline, women with POAF tend to have more comorbidities than their male counterparts. A systematic literature review and meta-analysis (n=14,970 patients) found that following cardiac surgery, females who experienced POAF had nearly double the risk of in-hospital mortality as males, although this differential in mortality risk did not persist after discharge [104]. POAF in females has also been associated with longer hospital stays [86]. Further studies are needed, particularly with respect to race and ethnicity combined with sex.

Discussion

Despite better monitoring tools and greater awareness, the rates of POAF have not changed appreciably over the decades. Moreover, there is no reason to think that POAF will decrease in the future: the inversion of the age pyramid, the obesity epidemic, and higher surgical volumes will all contribute to more cases of POAF. 

This lack of a consensus definition may be explicable because POAF may not be a single entity. POAF following cardiac surgery seems to differ mechanistically from POAF after noncardiac surgery, and there may even be differences between the so-called “early” versus “late” forms of POAF. The rate and implications of POAF following oncologic surgery have only recently emerged and may be of great importance as cancer rates increase. Racial disparities exist and seem explicable mainly by genetics, while sex differences are less explicable.

The potential dangers of postoperative atrial flutter, more common in Black patients than White patients, are not yet fully known. Some studies have grouped preoperative atrial flutter with POAF, but these two supraventricular tachyarrhythmias are mechanistically different. Of course, other studies do not even consider postoperative atrial flutter as a potential perioperative complication.

Some studies have limited POAF to an arrhythmia that occurs within a 30-day window following surgery, while others define POAF as an arrhythmia in the first few days after surgery [98]. The timing of the onset of POAF may provide clues as to the mechanisms of the arrhythmia.

POAF places an enormous burden on the healthcare system, in particular with respect to readmissions. The 30-day hospital readmission rate following CABG is 16.1%, with the most frequent diagnosis being AF (26.7%) [105]. Many explanations for this have been explored: socioeconomic disparities, health insurance status, access to care, lifestyle, risk factors such as age, sex, and comorbidities, genetic factors, including race and ethnicity, and cardiac anatomy. To some extent, all of these may play a role. In a retrospective study, individuals with Medicare or Medicaid had significantly higher 30-day readmission rates (18.4% and 20.2%, respectively) than those with private insurance (11.7%, p<0.0001) [105].

The role of race and sex in POAF epidemiology may hold important clues to its pathogenesis. Black patients who undergo CABG have lower incidences of POAF but more severe comorbidities at baseline compared to White patients [105]. Anatomical, biological, and genetic differences have been proposed [106]. It has also been speculated that more elusive forms of AF, such as sudden-onset asymptomatic short-duration episodes, might be more prevalent among Black patients than White patients [106]. Limited access to healthcare has been posited as a plausible explanation for this racial disparity, but in studies where all participants had identical forms of automatic monitoring, Black patients still had lower rates of AF than White patients. The various paradoxical findings related to POAF are summarized in Table 1.

**Table 1:** Paradoxical findings about POAF warrant further investigation POAF: postoperative atrial fibrillation; AF: atrial fibrillation References: Black paradox [7, 82], minority paradox [76, 80, 83], atrial flutter [94], and female paradox [103, 104]

	Paradox	Implications
Black paradox	Black patients have greater risks for POAF on average, but have lower rates of POAF. However, when POAF occurs, Black patients have worse outcomes.	Black patients also have lower rates of AF than other races despite having more and more severe risk factors. However, POAF is a significant predictor of mortality in Black patients.
Minority paradox	In the United States, minorities as a group have more risk factors for POAF and are less likely to have health insurance, but have less POAF than White patients.	The Black paradox may extend to other groups as well, indicating that White patients are a high-risk group.
Atrial flutter	Based on pacemaker data, Black patients had lower rates of AF but higher rates of atrial flutter compared to White patients. Atrial flutter can occur in the postoperative period, but has not been well studied.	Atrial flutter is a less common supraventricular tachyarrhythmia, but occurs more often in Black than White patients.
Female paradox	Controlling for other risk factors, females are less likely than males to develop POAF, but if POAF occurred in a female, she had a higher risk of long-term mortality than her male counterpart.	Called the “sex disparity,” females with POAF had almost double the risk of in-hospital mortality than their male counterparts; this risk was not durable post-discharge. Females with POAF also have longer lengths of stay than males with POAF.

This study has several limitations. It is a narrative review and not a systematic review based on a single research question that could be addressed by the results of clinical studies. The definition of POAF and atrial flutter used in the literature included in this study was not uniform, and our review reflects a diversity of these definitions. While it is clear that POAF can have long-term consequences, there are few studies on this phenomenon. The racial and sex paradoxes described here are well known to investigators but not elucidated, and seem to pose more questions than answers. Finally, our study is a narrative review of the literature and not a systematic analysis of a specific research question that can be answered with clinical trial results. Rather, it is an overview of our current understanding of the topic, open questions, knowledge gaps, and areas of consensus. It should not be confused with a systematic review and meta-analysis. 

## Conclusions

POAF is a common complication following surgery and occurs more often following cardiac surgery, particularly CABG and/or valve surgeries, and is more prevalent in men, geriatric patients, and those with underlying health conditions, such as cancer, sleep-disordered breathing, obesity, and heart disease, among others. While POAF may self-terminate without treatment, it should not be regarded as benign since it may be associated with long-term morbidity or mortality. Racial and ethnic differences have been perplexing since Black patients have more risk factors for POAF than White patients, but POAF occurs more often in White patients than in Black patients. However, when POAF does occur, Black patients have worse outcomes than Whites. While this paradox may be explained, at least in part, by genetics, a similar paradox has been observed in female versus male patients. Overall, a consensus definition of POAF is urgently needed to facilitate epidemiologic studies. Better surveillance and monitoring would also help better define the true incidence of POAF and its mid-term and long-term consequences.
